# ECCB 2026: the 25th European Conference on Computational Biology

**DOI:** 10.1093/bioinformatics/btag404

**Published:** 2026-08-21

**Authors:** Niko Beerenwinkel, Anne-Florence Bitbol, Natalia Gonzalez Gaarslev, Solange Guye

**Affiliations:** Department of Biosystems Science and Engineering, ETH Zurich, Basel, Switzerland; SIB Swiss Institute of Bioinformatics, Lausanne, Switzerland; SIB Swiss Institute of Bioinformatics, Lausanne, Switzerland; Institute of Bioengineering, School of Life Sciences, EPFL, Lausanne, Switzerland; SIB Swiss Institute of Bioinformatics, Lausanne, Switzerland; SIB Swiss Institute of Bioinformatics, Lausanne, Switzerland

## 1 Introduction

This volume of Bioinformatics includes the proceedings papers of the 25th European Conference on Computational Biology (ECCB 2026), taking place in Geneva, Switzerland, from 31 August to 4 September 2026, under the theme “Biodiversity, AI & Health: computational biology to address the challenges of our time.” The conference is organised by the SIB Swiss Institute of Bioinformatics, the national organisation for biomedical and biological data in Switzerland, with the support of the International Society for Computational Biology (ISCB). The conference will be hosted at the International Conference Centre Geneva (CICG). More information on ECCB 2026 is available at https://eccb2026.org.

ECCB is one of the leading international conferences in computational biology and bioinformatics, alongside the Intelligent Systems for Molecular Biology (ISMB) and the Research in Computational Molecular Biology (RECOMB) conferences. ECCB is organised jointly with ISMB in odd-numbered years and independently in even-numbered years.

The 2026 scientific programme covers methodological advances and applications in computational biology across five broad scientific areas:

1. Genomics, epigenomics and genome editing
2. Transcriptomics and gene regulation
3. Proteins and structural biology
4. Systems biology, multi-omics integration and modelling
5. Biodiversity, sustainability and environmental bioinformatics

It includes proceedings talks presenting original unpublished research, highlight talks featuring recently published work, poster sessions, tutorials and workshops, ELIXIR community sessions, focus sessions on emerging topics, industry-sponsored talks, and community-led satellite activities.

The conference features keynote lectures by distinguished invited speakers, namely Jeremy Farrar (World Health Organisation (WHO), Switzerland), Stephen Quake (Stanford University, USA), Anna-Sapfo Malaspinas (University of Lausanne and SIB, Switzerland), and Aleksandra Walczak (CNRS, France). Swiss President Guy Parmelin will open the conference. Invited focus sessions will explore transformative computational biology approaches for large-scale genomics and data infrastructures, as well as biodiversity informatics and big data frameworks for climate and earth system research.

ECCB 2026 also includes public engagement activities exploring the contribution of biological and biomedical data to society and the economy (see ‘Public engagement’ section below).

## 2 Proceedings

ECCB 2026 received 275 full manuscript submissions for proceedings talks from 37 countries. After desk rejections and 2 withdrawals, 267 manuscripts were reviewed. Submissions were assigned to one of the five scientific areas according to the authors’ preferences and the scientific content of the paper, with adjustments where needed to balance areas and manage conflicts of interest.

The review process was overseen by the Scientific Committee Chairs, Niko Beerenwinkel and Anne-Florence Bitbol, together with the 21 Area Chairs listed in Table 1. Each manuscript was evaluated by at least three reviewers (see Supplementary Material), and most manuscripts received four independent reviews. Overall, the 267 submissions that entered full review received an average of 3.91 reviews per paper, with a total of 1045 returned reviewer reports.

**Table 1:** Scientific areas of ECCB 2026 proceedings submissions.

Scientific areas	*Area chairs* and co-chairs	Submissions	Accepted		Percent accepted (%)
1. Genomics, epigenomics and genome editing	*Knut Reinert*, Maria Anisimova, Mohammed El-Kebir, Shilpa Garg	57	13		22.8
2. Transcriptomics and gene regulation	*Uwe Ohler*, Julien Gagneur, Raphaëlle Luisier, Magnus Rattray	61	11		18.0
3. Proteins and structural biology	*Ivet Bahar*, Elodie Laine, Emmanuel Levy, Basile Wicky	60	12		20.0
4. Systems biology, multi-omics integration and modelling	*Alfonso Valencia*, Anaïs Baudot, Valentina Boeva, María Rodríguez Martínez	62	11		17.7
5. Biodiversity, sustainability, and environmental bioinformatics	*Katja Bärenfaller*, Laurent Jacob, Avidan U. Neumann, Lucas Paoli, Sanja Vickovic	33	7		21.2
		273	54		19.8

The review process was supported by ISCB using the EasyChair conference management system (www.easychair.org). In addition to scientific excellence, reviewers were also asked to assess reproducibility, specifically code and data availability, where applicable. Following the review phase, reviewers had the opportunity to discuss submissions and refine their evaluations. Area Chairs facilitated these discussions and prepared recommendations in consultation with the Scientific Committee Chairs.

**Figure 1:**
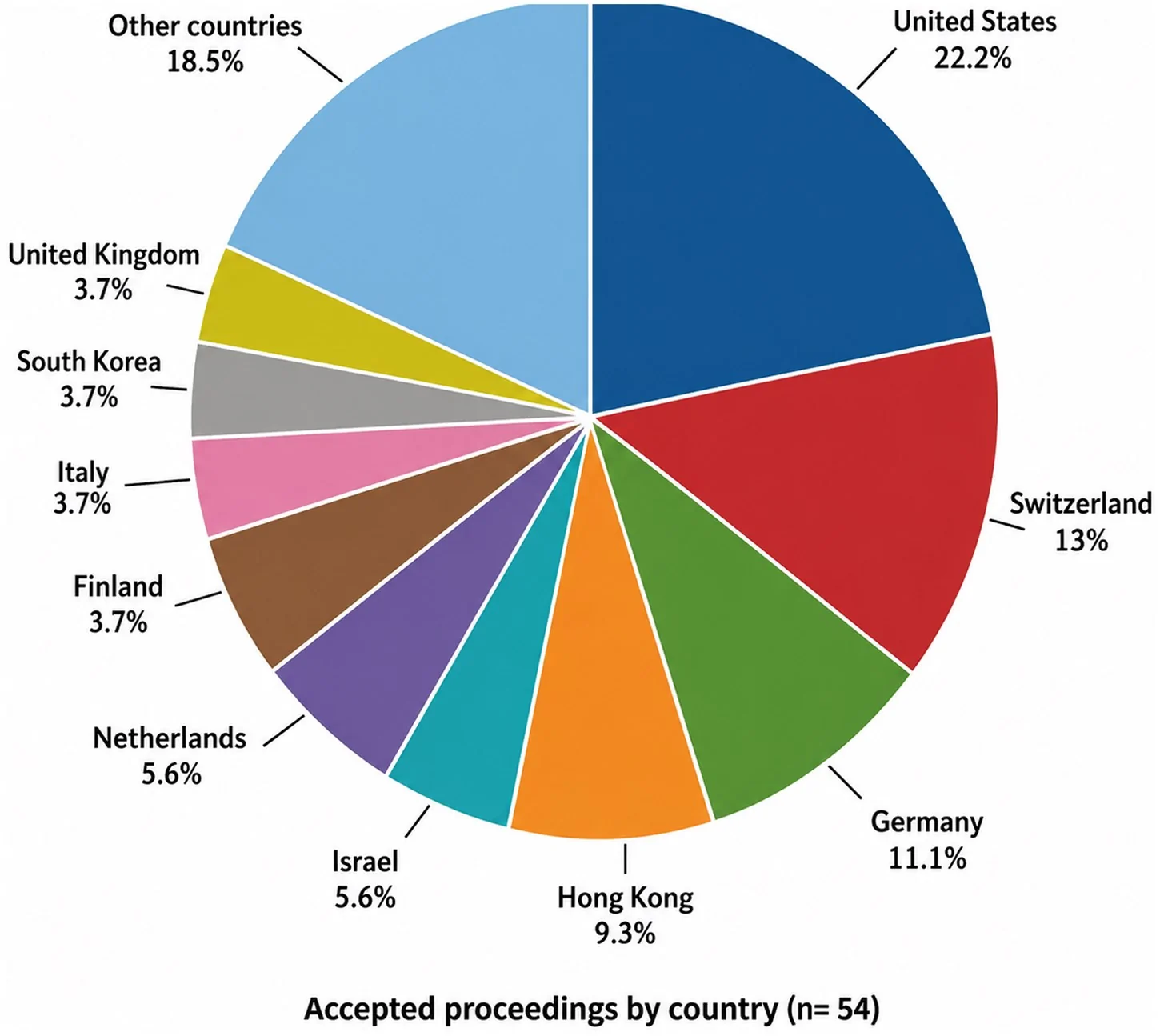
Distribution of accepted proceeding papers by country or region of the presenting author’s primary affiliation. Countries accounting for less than 2% of accepted papers were grouped under “Other countries.”

After completion of the revision process, 54 papers were accepted for presentation at ECCB 2026 and publication in the conference proceedings, corresponding to an overall acceptance rate of 19.8% of submitted manuscripts (Table 1). The countries or regions with the highest number of accepted Proceedings papers at ECCB 2026 were the United States, Switzerland, Germany, and Hong Kong, jointly contributing to more than half of the accepted papers (Fig. 1). Israel, the Netherlands, Finland, Italy, South Korea, and the United Kingdom also contributed a substantial number of accepted papers, highlighting the broad international representation of the conference.

### 2.1 Conflict-of-interest policy

ECCB 2026 followed a strict conflict-of-interest policy throughout the review and selection process. Submissions for which an Area Chair had a conflict of interest were handled separately by other non-conflicted Area Chairs. Care was also taken not to assign papers to reviewers with conflicts of interest. Conflicts of interest were defined as including recent collaborations, same institutions, advisor–advisee relationships, family or close personal relationships, direct competition, or any other circumstances that could interfere with objective evaluation. The Scientific Committee Chairs oversaw the management of conflict-of-interest cases to ensure fairness and confidentiality during the review process.

## 3 Highlight talks

The ECCB 2026 call for Highlight Talks invited presentations of recent work published or accepted for publication in peer-reviewed venues. The call received 223 submissions, of which 147 passed the initial pre-screening for eligibility and entered peer review. Submissions were ranked according to their relevance and impact in computational biology, and their suitability for presentation to a broad ECCB audience. The Area Chairs (listed in Table 1) selected 28 highlight talks for presentation at the conference, corresponding to an acceptance rate of 19.0% of the reviewed submissions.

## 4 Posters

ECCB 2026 received over 800 poster submissions, with a large majority of submissions accepted  for presentation and distributed across three poster sessions. Poster sessions provide participants with the opportunity to present recent and ongoing work, exchange ideas, and foster collaborations across the conference’s scientific areas.

## 5 Tutorials, workshops and Student Council Symposium

Tutorials and workshops will take place on 3 September 2026, alongside the ISCB Student Council Symposium. ECCB 2026 received 47 tutorial and workshop proposals, of which 17 were selected. Following one withdrawal, the final programme comprises 13 tutorials and 3 workshops. In addition, two workshops organised by ELIXIR, one of the ECCB 2026 sponsors, were selected through a separate internal process.

The review and selection process was coordinated by Diana Marek and Patricia Palagi, Chairs of the Tutorials and Workshops Committee, together with a committee of nine members (Rasool Saghaleyni, Olivier Sand, Farzana Rahman, Xavier Robin, Ragothaman Yennamalli, Joana Carlevaro, Deepak Tanwar, Geert van Geest, and Wandrille Duchemin). Each submission was evaluated by three independent reviewers.

Tutorials and workshops provide participants with opportunities for hands-on training, practical exchange, and discussion of emerging topics in computational biology and bioinformatics.

The conference also hosts the ISCB Student Council Symposium, organised by and for early-career researchers.

## 6 ELIXIR and Communities Day

ECCB 2026 includes an ELIXIR community track, with four dedicated sessions highlighting developments in European life-science data infrastructures, standards, services, and community-driven resources.

The conference will also host a Communities Day on 4 September 2026, bringing together SIB- and community-led initiatives around topics such as biodiversity, pathogen surveillance, research infrastructures and data-intensive life sciences.

## 7 Public engagement

In line with the conference theme and Geneva’s role as an international hub for science and policy, ECCB 2026 will foster dialogue between scientists, policymakers and the public through a public engagement programme on the contributions of biological and biomedical data to society and the economy. It comprises:

- A month-long open-air exhibition ahead of ECCB 2026. Starting on August 3, 2026, the DATARIUM exhibition will showcase how biological and biomedical data contribute to advances in health, biodiversity and trustworthy AI through large-scale visual installations in public spaces (https://datarium.ch). To enrich the experience, guided tours will be offered to high school classes during the second half of August.
- A Public Forum bringing together science, society and policy. Held on the conference opening day, August 31, 2026, the event will combine short scientific talks, interactive workshops, and a roundtable discussion on responsible AI, data sovereinty and global competition (https://genevalovesdata.org/forum-public).

## 8 Diversity, inclusion and conduct

ECCB 2026 is committed to creating a safe, respectful, and inclusive environment for all participants. The conference Code of Conduct sets out expected standards of behaviour and procedures for reporting concerns. The organisers have also promoted diversity and gender balance across committees and invited speakers, following practices highlighted in previous ECCB editorial briefs.

## Supplementary Material

btag404_Supplementary_Data

## Data Availability

All data are incorporated into the article. Additional information is available on the ECCB 2026 conference website at https://eccb2026.org.

