# Supplementary material for: ECCB 2026: the 25th European Conference on Computational Biology
Source: Bioinformatics. 2026 Aug 21;42(Suppl 2):btag404. doi: 10.1093/bioinformatics/btag404 (PMC13501304; doi:10.1093/bioinformatics/btag404)
Supplement: Supplementary file 1 [file btag404_supplementary_data.pdf]

ECCB 2026 Committees

PROGRAM COMMITTEE

Program Committee Chair:
Christophe Dessimoz | SIB Swiss Institute of Bioinformatics Executive Director and Group Leader, Comparative Genomics, CH

Anne-Florence Bitbol | SIB Swiss Institute of Bioinformatics
Group Leader, Laboratory of Computational Biology and Theoretical Biophysics EPFL, Lausanne, CH

Niko Beerenwinkel | SIB Swiss Institute of Bioinformatics
Group Leader, Computational Biology Group, ETH Zurich, D-BSSE Basel, CH

Diana Marek | Training Manager & Computational Biologist, SIB Swiss Institute of Bioinformatics, CH

Patricia Palagi | Director, Training SIB Swiss Institute of Bioinformatics, CH

Robert Waterhouse | SIB Swiss Institute of Bioinformatics Group Leader, Environmental Bioinformatics, CH

SCIENTIFIC COMMITTEE

Scientific Committee Chairs:

Anne-Florence Bitbol | SIB Group Leader, Laboratory of Computational Biology and Theoretical Biophysics EPFL, Lausanne, CH

Niko Beerenwinkel | SIB Group Leader, Computational Biology Group, ETH Zurich, D-BSSE Basel, CH

Area chairs and co-chairs :

Genomics, epigenomics and genome editing

Area Chair**: Knut Reinert** | Free University of Berlin, DE
Co-chairs:
Maria Anisimova | ZHAW, Institute of Applied Simulation / SIB Swiss Institute of Bioinformatics, CH
Mohammed El-Kebir | University of Illinois at Urbana-Champaign, USA
Shilpa Garg | Manchester University, UK

Transcriptomics and gene regulation

Area Chair: **Uwe Ohler** | Max Delbruck Center, Humboldt University, DE
Co-chairs:
Raphaëlle Luisier | University of Bern/SIB Swiss Institute of Bioinformatics, CH
Magnus Rattray | Manchester University, UK
Julien Gagneur | TUM Technische Universität München, DE

Proteins and structural biology

Area Chair: **Ivet Bahar** | Stony Brook University, NY, US / Laufer Center for Physical & Quantitative Biology
Co-chairs:
Elodie Laine | Sorbonne University, FR
Emmanuel Levy | University of Geneva, CH
Basile Wicky | ETH Zurich, CH

Systems biology, multi-omics integration and modelling

Area Chair: **Alfonso Valencia** | Barcelona Supercomputing Center, ES
Co-chairs:
Anaïs Baudot | Aix-Marseille University, FR
Valentina Boeva | ETH Zurich / SIB Swiss Institute of Bioinformatics, CH
María Rodríguez Martínez | Yale School of Medicine, US

Biodiversity, sustainability, and environmental bioinformatics

Area Chair: **Katja Bärenfaller** | SIAF and University of Zurich / SIB, CH
Co-chairs:
Laurent Jacob | CNRS, Sorbonne Univ., FR
Avidan U. Neumann | University of Augsburg and Helmholtz Munich, DE
Lucas Paoli | EPFL, CH
Sanja Vickovic | Columbia University, US

PROCEEDINGS COMMITTEE REVIEWERS

Genomics, epigenomics and genome editing

Aaron Pfennig
Alyssa Morrow
Ana Castillo Orozco
Andre Kahles
Antoine Limasset
Anton Feenstra
Irene Julca
Ivan Ovcharenko
Jacques Fellay
Jasmijn Baaijens
Jens Stoye
Jens-Uwe Ulrich
Palash Sashittal
Panagiotis Chouvardas
Pranam Chatterjee
Rainer Spang
Ralf Herwig
Ralf Zimmer

Bernhard Haubold
Bernhard Renard
Birte Kehr
Brona Brejova
Can Alkan
Cedric Chauve
Christophe Klopp
Costas Bouyioukos
Cristian Groza
Daniel Huson
David Koslicki
Endre Barta
Francisco M De La Vega
Gaetan De Waele
Gonga Ma
Haoyu Cheng
Henrik Latz
Hoon Cho
Karel Brinda
Katharina Jahn
Kay Nieselt
Lars Kaderali
Louis du Plessis
Magnus Palmblad
Markus Bauer
Martin Vingron
Matthias Zytnicki
Michael Charleston
Mikael Marttinen
Mikhail Kolmogorov
Mohamed Abouelhoda
Nadège Guiglielmoni
Nadia El-Mabrouk
Nadia Pisanti
Natasha Glover
Nico Pfeifer
Raluca Uricaru
Ritambhara Singh
Russell Schwartz
Salem Malikic
Sebastian Böcker
Simone Zaccaria
Solon Pissis
Stefano Lonardi
Tatsuya Akutsu
Tibo Vande Moortele
Tobias Marschall
Tobias Rausch
Uthsav Chitra
Veli Mäkinen
Victoria Popic
Vinicius Maracaja-Coutinho

Transcriptomics and gene regulation

Alexander Sasse
Alicia Oshlack
Andreas J. Gruber
Anna R. Poetsch
Annalisa Marsico
Anob Chakrabarti
Anthony Baptista
Anthony Mathelier
Arnaud Liehrmann
Bartek Wilczynski
Ben Raphael
Borbala Misfud
Brian Clarke
Can Firtina
Caroline Friedel
Carsten Marr
Charlotte Capitanchik
Chloe Steen
Eivind Valen
Florian Buettner
Gioele LaManno
Giulio Caravagna
Guanao Yan
Guido Sanguinetti
Harri Lähdesmäki
Helene Kretzmer
Ivan G. Costa
Ivo Grosse
Jan Grau
Jan Jakub Zylicz
Jesper Tegner
Joana Carlevaro
Judith Zaugg
Laleh Haghverdi
Leelavati Narlikar
Lonnie Welch
Manfred Claassen
Manja Marz
Marianna Rapsomaniki
Mark Robinson
Martin Kircher
Matthias Heinig
Michael Bockmayr
Mihaela Modic
Mor Nitzan
Mudassar Iqbal
Panagiotis Alexiou
Philipp Bucher
Ramana Davuluri
Robin Dowell
Rolf Backofen
Sai Zhang
Shaun Mahony
Silvio Bicciato
Smita Krishnaswamy
Sokratia Georgaka
Stefan Canzar
Sushmita Roy
Sven Rahmann
Takis Benos
Teresa Przytycka
Virpi Ahola
William Majoros
William Noble
Yaron Orenstein
Yuanhua Huang
Yves Moreau

Proteins and structural biology

Alena Khmelinskaia
Alexander Monzon
Anna Marabotti
Anne-Claude Camproux
Antoine Taly
Arjun Ray
Attila Gursoy
Bjorn Wallner
Canan Atilgan
Chan Cao
Chris Kieslich
Christina Boucher
Daisuke Kihara
Didier Barradas-Bautista
Diego Zea
Douglas Pires
Edoardo Sarti
Florence Tama
Florian Praetorius
Frances Pearl
Frauke Gräter
Frederic Cazals
Frédérique Lisacek
George Spyrou
Janani Durairaj
Janet Thornton
Jean-Christophe Gelly
Joana Pereira
Juan Fernandez-Recio
Kliment Olechnovic
Laura Orellana
Lee-Wei Yang
Liisa Holm
Maria Freiberger
Markus Lill
Martin Pacesa
Martin Weigt
Matteo Dal Peraro
Mehrnoosh Arrar
Michael Nilges
Oznur Tastan
Paolo De Los Rios
Pierre Barrat-Charlaix
R. Gonzalo Parra
Raphaël Guerois
Ravinder Abrol
Robert Jernigan
Rohit Singh
Samuela Pasquali
Sergei Grudinin
Slavica Jonic
Stefano Vanni
Sucharita Dey
Tatiana Galochkina
Thérèse Malliavin
Thomas Hamelryck
Thomas Lemmin
Victor Greiff
Vincent Mallet
Yang Lu
Yasaman Karami

Systems biology, multi-omics integration and modelling

Alberto Valdeolivas
Alexandros Xenos
Ana Rojas
Andreas Zanzoni
Andrée Delahaye
Anthony Gitter
Blerina Sinaimeri
Carito Guziolowski
Carme Zambrana
Cassandra Burdziak
Cenk Sahinalp
Chad Myers
Charles Lecellier
Christine Brun
Christos Ouzounis
Clémence Frioux
Clémence Réda
Denis Thieffry
Devon Kohler
Fatima Al-Shahrour
Fatima Sanchez Cabo
Florencio Pazos
Franzisca Singer
Galadriel Brière
Hidde De-Jong
Ioannis Xenarios
Joaquim Aguirre-Plans
Joaquin Dopazo
John Lees
Jon Sánchez Valle
Jörg Stelling
Juan Nogales
Julien Chiquet
Katharina Baum
Kimberly Glass
Lauren Cowley
Laurence Calzone
Lenore Cowen
Maxime Folschette
Miguel Rocha
Miquel Duran-Frigola
Nicola Zamboni
Nicolas Sundqvist
Nurcan Tuncbag
Olivier Dameron
Patricia Thebault
Paul Villoutreix
Pierre Larmande
Roded Sharan
Ron Shamir
Simona Cristea
Solene Song
Steve Hoffmann
Trey Ideker
Ugo Bastolla
Vassily Hatzimanikatis
Vincent Noël

Dirk Husmeier
Elias Ventre
Emmanuelle Becker
Enrico Glaab
Erik van Nimwegen
Loïc Paulevé
Luis Rocha
Macha Nikolski
Marco Ruscone
Maria Chikina
Xiuwei Zhang
Yuna Blum

Biodiversity, sustainability, and environmental bioinformatics

Agnes Kiragga
Alan Pacheco
Alexandros Stamatakis
Amélie Leroy
Anna Sintsova
Anna Zhukova
Annabelle Haudry
Anne Siegel
Anne-Louise Leutenegger
Antoine Szatkownik
Athina Gavriilidou
Bastien Boussau
Ben J. Woodcroft
Burak Yelmen
Carl Herrmann
Chris Quince
Claude Pasquier
Claudia Traidl-Hoffmann
Denise Kühnert
Devlina Chakravarty
Edoardo Pasolli
Flora Jay
Franz Baumdicker
Frederic Bastian
Guillaume Gautreau
Guillem Salazar
Ignacio Quintero
Jaime Huerta-Cepas
Karoline Faust
Karthik Anantharaman
Lois Maignen
Luis Pedro Coelho
Luise Rauer
Manolo Perez
Marc Robinson-Rechavi
Maria Plaza
Marija Dmitrijeva
Marissa Kosnik
Matteo Fumagalli
Matthew Olm
Nathalie Vialaneix
Nelle Varoquaux
Richard Neher
Rob D. Finn
Roland Faure
Sakina-Dorothée Ayata
Samuel Miravet Verde
Serina Robinson
Sofia Paraskevopoulou
Tandy Warnow
Tess Brewer
Thankgod Ebenezer
Thibault Latrille
Thomas S.B. Schmidt
Tim Roloff Handschin
Victoria Bourgeais
Yann Le Cunff
Yosuke Nishimura

WORKSHOPS & TUTORIALS COMMITTEE

Chairs:
Diana Marek | Training Manager & Computational Biologist, SIB Swiss Institute of Bioinformatics, CH
Patricia Palagi | Director, Training SIB Swiss Institute of Bioinformatics, CH

Rasool Saghaleyni | SciLifeLab (Sweden)
Olivier Sand | CNRS, FR
Farzana Rahman | Kingston University London, UK
Xavier Robin | SIB Swiss Institute of Bioinformatics, CH
Ragothaman Yennamalli | SASTRA Deemed University (India)
Joana Carlevaro | SIB Swiss Institute of Bioinformatics, CH
Deepak Tanwar | SIB Swiss Institute of Bioinformatics, CH
Geert van Geest | SIB Swiss Institute of Bioinformatics, CH
Wandrille Duchemin | SIB Swiss Institute of Bioinformatics, CH

ORGANISING COMMITEE

Marie Dangles | Director, External & Internal Relations, SIB Swiss Institute of Bioinformatics, CH
Elsa Dubar | Communications & Event Manager, SIB Swiss Institute of Bioinformatics, CH
Natalia González Gaarslev | Events Manager, SIB Swiss Institute of Bioinformatics, CH
Solange Guye | Scientific Events Manager, SIB Swiss Institute of Bioinformatics, CH

POSTERS COMMITTEE

Anna Parker | SIB Swiss Institute of Bioinformatics, CH
Antoine Daina | SIB Swiss Institute of Bioinformatics, CH
Christian de Guttry | SIB Swiss Institute of Bioinformatics, CH
Frederic Schutz | SIB Swiss Institute of Bioinformatics, CH
Helena Seth Smith | University of Zurich, CH
Hubert Rehrauer | University of Zurich, CH
Janna Hastings | Idiap Research Institute, CH
Laurent Falquet | University of Fribourg, CH
Luciano Cascione | Institute of Oncology Research, CH
Marija Buljan | Swiss Federal Laboratories for Materials Science and Technology, CH
Mark Robinson | University of Zurich, CH
Marta Andreia Da Silva Perez Gomes | SIB Swiss Institute of Bioinformatics, CH
Michael Baudis | University of Zurich, CH
Nadine Fournier | SIB Swiss Institute of Bioinformatics, CH
Patrick Ruch | SIB Swiss Institute of Bioinformatics, CH
Robert Ivanek | University of Basel, CH
Ute Roehrig | SIB Swiss Institute of Bioinformatics, CH
Witold Wolski | University of Zurich, CH
